# Experimental Study on Ultrasonic-Assisted End Milling Forces in 2195 Aluminum-Lithium Alloy

**DOI:** 10.3390/ma15072508

**Published:** 2022-03-29

**Authors:** Hongtao Wang, Shaolin Zhang, Guangxi Li

**Affiliations:** 1 School of Mechanical and Power Engineering, Zhengzhou University, Zhengzhou 450001, China; wanghongtaozzu@163.com (H.W.); zhangshaolin@zzu.edu.cn (S.Z.); 2 Henan Engineering Research Center for Ultrasonic Technology and Application, Pingdingshan University, Pingdingshan 467000, China

**Keywords:** 2195 Al-Li alloy, ultrasonic milling, machining parameters, milling force, surface appearance

## Abstract

To achieve high-quality machining of the 2195 aluminum-lithium alloy, this paper presents an experimental study on the effect of milling processing parameters on milling forces and surface topography, during which conventional milling and longitudinal-torsional ultrasonic vibration milling of the 2195 Al-Li alloy were performed. The characterization of the tool tip trajectory illustrates some of the advantages of ultrasonic machining, which include variable depth of cut and tool chip pulling. The differences in milling forces between conventional milling and longitudinal-torsional ultrasonic vibration machining were compared using orthogonal tests, and the effect of ultrasonic vibration on milling forces was investigated in detail. The maximum reduction of milling force *Fy* in the feed direction under the influence of torsional vibration is 62% and 54% for larger feed per tooth and cutting depth, respectively. The high-frequency impact generated by the longitudinal vibration not only reduces the chip accumulation on the surface, but also smooths out the tool-tooth scratches and creates a regular surface profile. In addition, the characteristics of the milling force signals of the two machining methods were analyzed, and the analysis of the spectrum of the collected milling forces revealed that the ultrasonic vibration caused the high-frequency components of the milling forces *Fy* and *Fz*. The orthogonal result analysis and single-factor result analysis verified the superiority of ultrasonic machining, provided parameter selection for subsequent aluminum-lithium alloy machining, and bridged the gap of longitudinal torsional ultrasonic vibration machining of 2195 aluminum-lithium alloy in the study of milling force.

## 1. Introduction

Aluminum-lithium alloys are lightweight aerospace structural materials with excellent comprehensive properties such as low density, low weight, high elastic modulus, high specific strength and specific stiffness [1,2,3]. Among them, 2195 aluminum alloy is one of the most complex grades in the wrought aluminum-copper family (2000 or 2xxx series), with at least 91.9% aluminum by weight. The high strength-to-weight ratio of this alloy made it lucrative for aerospace applications. Thus, it successfully replaced the 2219 alloy in the Space Shuttle fuel outer storage tank, reducing its mass by 5% and increasing the storage capacity by 3.4 t [4]. More recently, 2195 alloy was used for the propellant tanks of the Falcon 9 Full Thrust orbital launcher and the new ULA Vulcan first stage.

Like most other aluminum-copper alloys, 2195 is a high-strength alloy, with bad workability and poor corrosion resistance. As a wrought alloy, it can be welded, particularly by friction stir welding, and is fracture resistant at cryogenic temperatures. Alternatively, the processing of aluminum-lithium alloys has been achieved by the chemical etching method [5,6,7,8,9], which was not environmentally-friendly due a series of pollution problems. Nowadays, mechanical milling has made great progress, which improves the milling efficiency and avoids environmental pollution.

The ease of combustion and oxidation of Mg and Li elements in 2195 Al-Li alloy leads to increased difficulty in thermomechanical treatment and poor machinability of Al-Li alloy under high-speed milling [10]. The study of processing methods and process parameters [11] of aluminum-lithium alloy products is a good guide for engineering applications. Mou et al. [12] studied the surface integrity of aluminum-lithium alloys obtained by milling under different coolant conditions, and the results showed that the milling direction and the angle of contact between the milling tool and the test piece have a large effect on the surface roughness. Rong et al. [13] studied the surface roughness of aluminum-lithium alloys using high-speed milling and investigated the variation law of process parameters on surface roughness. So far, the difficulty of machining aluminum-lithium alloys has diminished, but the biggest challenge remaining is how to achieve good surface integrity and ideal chip control by controlling the machining parameters [14]. Most researchers have focused more on the surface morphology of Al-Li alloys, while machining parameters often affect the variation of milling forces and change the surface morphology.

The magnitude of the milling force determines the power consumed during the milling process and the deformation of the machining system [15,16], while the machining parameters have a direct influence on the milling force and surface topography. Tej Pratap et al. [17] investigated the high-speed milling process by the finite element modeling (FEM) of the titanium alloy milling process, which showed that strain rate and temperature significantly influenced the cutting force. Sela et al. [18] established a model of milling force under high-speed milling of 7455-T7351 aluminum alloys and studied the predicted value of cutting force under orthogonal test. Girish et al. [19] designed an end mill tool for ultrasonic milling, based on the orthogonal test method for ultrasonic milling of the Al6063 aluminum alloy, and established regression equations to assess the cutting forces during ultrasonic milling. The results showed that the feed rate had the strongest effect on the cutting forces among other machining parameters.

Ultrasonic vibration machining techniques can reduce milling forces [20,21] and improve surface quality. In recent years, ultrasonic vibration machining has been gradually applied to alloys with poor machinability and to processes such as grinding and drilling of conformal materials [22,23], which provides a new idea for the machining of aluminum-lithium alloys. Compared with conventional machining, machining with longitudinal vibration coupled with torsional vibration can change the tool tip variation from linear to intra-dimensional space motion, thus further reducing the milling force and improving the machining quality [24,25]. So far, ultrasonic vibration processing has mostly focused on titanium alloys and other composite materials, while domestic and foreign scholars have not conducted longitudinal-torsional ultrasonic vibration milling of aluminum-lithium alloy materials. Therefore, it is necessary to study the longitudinal-torsional ultrasonic vibration on the milling force of the 2195 aluminum-lithium alloy.

This paper presents the results of an experimental study on the effects of machining parameters on milling forces and surface topography for conventional and longitudinal torsional ultrasonic vibration milling of the 2195 aluminum-lithium alloy. This study can fill the gap in the field of longitudinal ultrasonic vibration milling for the 2195 Al-Li alloy, thus laying the foundation for high-quality machining of the 2195 Al-Li alloy.

## 2. Material and Experiment Methods

### 2.1. Materials

Tests were performed on the 2195 Al-Li alloy (Ocean Power Rare Metal Products Co, Suzhou, China), which chemical composition and mechanical properties at room temperature are listed in Table 1 and Table 2, respectively. Workpieces used in all tests were made from the same sheet to prevent additional effects caused by different batches of various specifications. The sheet was cut into 15 mm × 7 mm × 8 mm blocks of specimens by wire cutting (Cyang, Guangdong, China).

### 2.2. Test Setup

**Figure 1:**
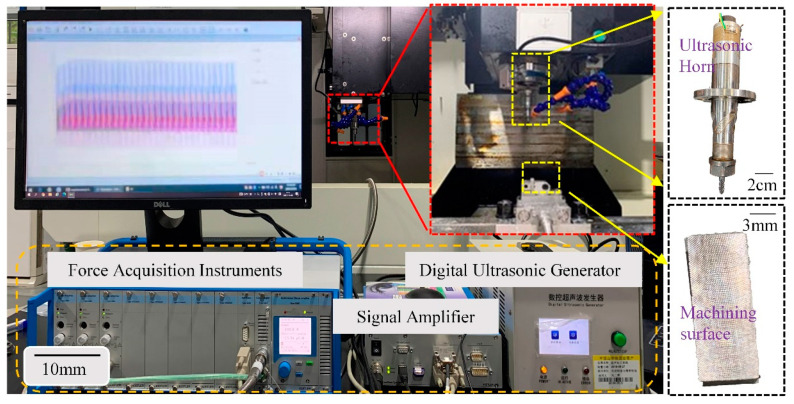
Longitudinal torsion ultrasonic-assisted milling test set.

The test setup for milling force testing is shown in Figure 1. A CNC machining center (Henfux-HFM 700L, Taiwan, China) with conventional milling and ultrasonic milling capabilities and a maximum rotational speed of 30,000 r/min was used in the tests. Its spindle includes a piezoelectric transducer, an ultrasonic transducer and a tool holder. The induction is generated by a transmitting coil fixed to the machine and a receiving coil rotating coaxially on the spindle to power the tool. Ultrasonic vibrations are applied to a piezoelectric transducer, which is to be amplified by a variable amplitude horn, resulting in longitudinal vibrations and torsional vibrations applied to the tool. To study the milling force of ultrasonic-assisted milling of the 2195 aluminum-lithium alloy, a Kistler 5080 piezoelectric crystal force meter (Kistler, Winterthur, Switzerland) was set to 150 kHz acquisition frequency and 150 N acquisition range. The milling force signal of longitudinal-torsional ultrasonic assisted milling (L-UVAM) was collected online in real-time scale, and the average milling forces in three directions (*x*, *y*, and *z*) during smooth cutting were recorded via the signal amplifier.

### 2.3. Tool-Workpiece Kinematics Analysis

To improve the material removal rate, a four-flute integral general-purpose carbide end mill (model: GM-4E-D8.0 R1.0, Zhuzhou cemented carbide Cutting Tools Co., Zhuzhou, China) was used. The milling cutter specifications were as follows: diameter of 8 mm, edge length of 20 mm, total length of 60 mm and coated with AlCrXN.

**Figure 2:**
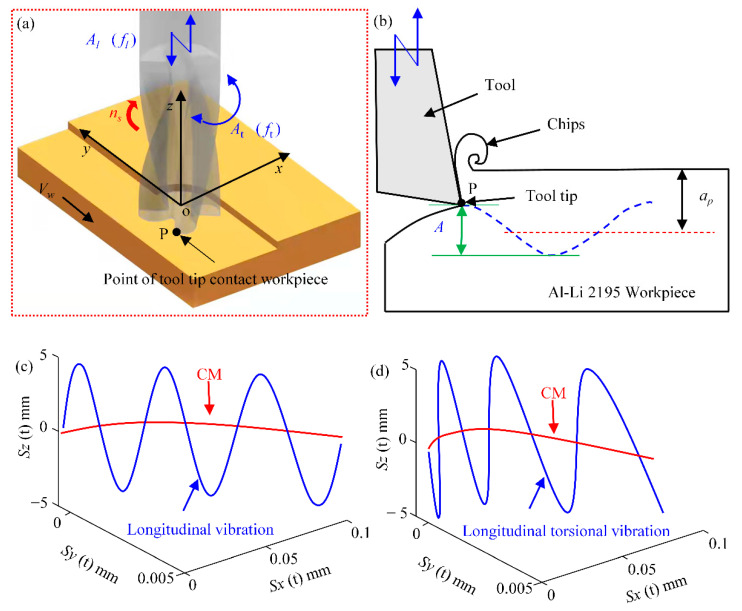
Schematic diagram of the variation lever and tool (**a**) Tool trajectory analysis; (**b**) Principle of Ultrasonic Vibration Milling; (**c**) Longitudinal ultrasonic vibration tool tip motion trajectory; (**d**) Longitudinal-torsional ultrasonic vibration tool tip motion trajectory.

Figure 2a shows the schematic diagram of longitudinal torsional ultrasonic vibration milling process. The analysis of the cutting edge trajectory during ultrasonic milling is essential for the analysis of milling forces in milling tests. The radius of the tool is *R*, the tool tooth number is N, and the coordinate system *O-xyz* is established between the tool and the center point of the surface contacted by the 2195 Al-Li specimen, where the point P on the edge of the end mill is used as the reference point. The x-direction is the radial direction, the y-direction is the tool feed direction, and the z-direction as the direction of applied ultrasonic vibration. We define *A* as the amplitude of ultrasonic vibration, *A_l_* as the amplitude of longitudinal vibration and *f_l_* as the frequency of longitudinal vibration. Accordingly, *A_t_* is the amplitude in the torsion direction and *f_t_* is the frequency in the torsion direction. The rotation speed of the spindle is *n_s_* and the speed in the feed direction is *v_w_*. The feed per tooth is *f_z_* and the depth of cut is *a_p_*.

Figure 2b shows the trajectory of the tool tip during milling, where s is the distance traveled by the tool tip. The trajectory of the reference point P with longitudinal ultrasonic vibration of the tool tip is shown in Figure 2c. The trajectory of the reference point P after the application of torsional torque is shown in Figure 2d. In longitudinal torsional ultrasonic vibration, the frequency and initial phase of both longitudinal and torsional vibration is identical, because the vibrations in both directions come together from the single amplitude. Thus, *f_l_* = *f_t_*, the ratio of amplitude in both torsional and longitudinal vibration is 0.6:1, and the initial phase *j* is the same. As a result, the equations of motion for conventional milling and longitudinal vibration are expressed by Equations (1) and (2), respectively. The trajectory of the point P in torsional vibration is shown in Equation (3). The trajectory of the longitudinal torsional vibration motion of the point P is shown in Equation (4).

(1) $$\left\{ \begin{array}{l} Sx(t)=R\sin\frac{2\pi n_{s}t}{60}\\ Sy(t)=Nfzt+R\cos\frac{2\pi n_{s}t}{60}\\ Sz(t)=0 \end{array}\right.$$

(2) $$\left\{ \begin{array}{l} Sx(t)=R\sin\frac{2\pi n_{s}t}{60}\\ Sy(t)=Nfzt+R\cos\frac{2\pi n_{s}t}{60}\\ Sz(t)=Al\sin(2\pi f_{l}t) \end{array}\right.$$

(3) $$\left\{ \begin{array}{l} Sx(t)=R\sin\left[\frac{2\pi n_{s}t}{60}+\frac{At\sin\left(2\pi f_{t}t+\varphi \right)}{R}\right]\\ Sy(t)=\frac{Nf_{z}n_{s}t}{60}+R\cos\left[\frac{2\pi n_{s}t}{60}+\frac{At\sin\left(2\pi f_{t}t+\varphi \right)}{R}\right]\\ Sz(t)=At\sin\left(2\pi f_{l}t+\varphi \right) \end{array}\right.$$

(4) $$\left\{ \begin{array}{l} Sx(t)=R\sin\left[\frac{2\pi n_{s}t}{60}+\frac{Al\sin\left(2\pi f_{l}t+\varphi \right)}{R}\right]\\ Sy(t)=\frac{Nf_{z}n_{s}t}{60}+R\cos\left[\frac{2\pi n_{s}t}{60}+\frac{Al\sin\left(2\pi f_{l}t+\varphi \right)}{R}\right]\\ Sz(t)=Al\sin\left(2\pi f_{l}t+\varphi \right) \end{array}\right.$$

According to the milling trajectories presented in Figure 2b,d, the path where the reference point P is located is a three-dimensional curve with “sinusoidal” fluctuations, while conventional milling is a simple two-dimensional curve. In actual machining, the longitudinal and torsional vibrations of the tool can form a series of vibration traces on the surface. Due to the variable tool acceleration, the cutting depth of the tool will also change. This machining feature reduces milling forces and creates a regular texture on the workpiece surface, optimizing surface integrity.

### 2.4. Test Program

The force gauge was turned on before the milling test, and the cutting fluid was not added during the test. The dynamometer was closed to terminate data collection when milling was complete. To ensure the accuracy of the milling force, the milling force signal was finally processed to acquire the mean value of the milling force.

Firstly, the test values of milling force under each machining parameter were analyzed by the method of an orthogonal test to acquire the results of machining parameter interaction. The polarization difference and variance of the machining parameters were then analyzed to finally determine the order of the effect of each machining parameter on the test results. The machining parameters that had the least effect on the results were used quantitatively, the machining parameters that had the greatest effect on the results were used as variables, and then single factor analysis of the milling force values was undertaken. The effect of milling force on the machined surface was obtained by observing the surface morphology after machining.

The test program is summarized in Table 3. During the test, there were many factors affecting the cutting force. Of these, four factors, namely milling speed *n_s_*, feed per tooth *f_z_*, cutting depth *a_p_* and amplitude *A*, were used in the test program.

## 3. Results and Discussion

The results of the orthogonal test on the milling force of the 2195 Al-Li alloy are presented in Table 4. A total of 27 sets of tests were conducted considering the interactive effects of milling speed, feed per tooth and ultrasonic amplitude. Since the diameter of the milling tool is larger than the end face of the workpiece, the entire surface can be milled in one tool feed. In the coordinate system *O-xyz*, the tool rotated counterclockwise, and the milling force *Fx* direction was along the opposite direction of the *x*-axis. The tool feeding in the opposite direction of the *y*-axis, the milling force *Fy*, is a positive number. Influenced by the longitudinal ultrasonic vibration, the “sinusoidal” motion of the tip of the tool and the workpiece had the characteristics of contact and separation, so there were positive and negative alternating phenomena of *Fz*. *Fx* and *Fy* did not alternate between positive and negative intervals because the workpiece width was much larger than the cutting depth.

### 3.1. Analysis of the Coupled Effect of Machining Parameters on the Milling Forces

**Figure 3:**
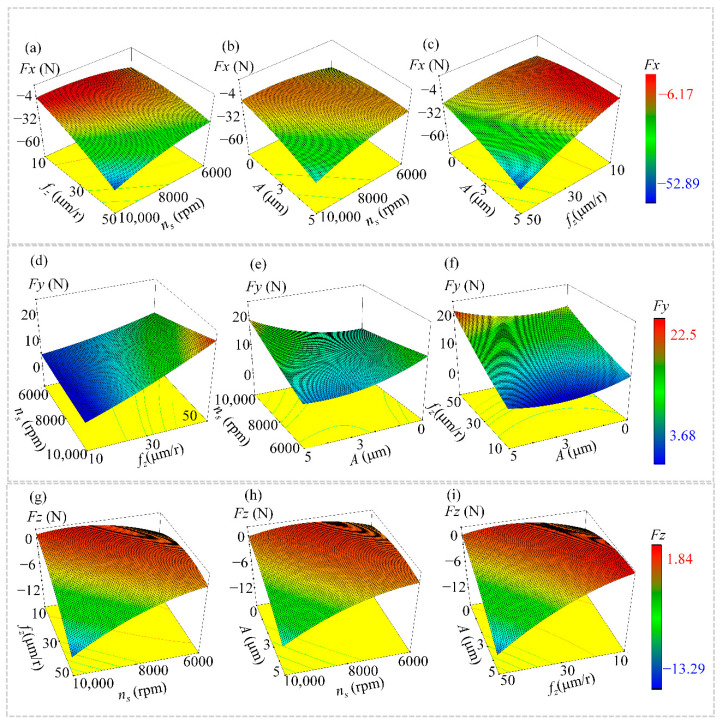
The coupled effects of milling parameters (*n_s_*, *f_z_* and *A*) on cutting forces *Fx*, *Fy* and *Fz*. (**a**,**d**,**g**) Interactive effect of feed per tooth and spindle speed on milling force *Fx*, *Fy*, *Fz*; (**b**,**e**,**h**) Interactive effect of ultrasonic amplitude and spindle speed on milling force *Fx*, *Fy*, *Fz*; (**c**,**f**,**i**) Interactive effects of ultrasonic amplitude and feed per tooth on milling forces *Fx*, *Fy*, *Fz*.

The interaction (coupling) effects of milling speed (*n_s_*), ultrasonic amplitude (*A*) and feed per tooth (*f_z_*) on the milling forces *Fx*, *Fy* and *Fz* are depicted in Figure 3. As seen in Figure 3a,d,g, a simultaneous increase in feed per tooth and rotational speed leads to a significant increase in the cutting forces *Fx*, *Fy* and *Fz*. At a high milling speed (*n_s_* = 8000 r/min) and using a smaller feed per tooth *f_z_* = 10μm/r can effectively reduce the milling force. At large feed per tooth (*f_z_* = 50 μm/r), the milling force can be reduced by decreasing the milling speed to *n_s_* = 6000 r/min.

The analysis of coupled effects in Figure 3b,e,h revealed that higher milling speeds weakened the effect of ultrasonic vibration, while large ultrasonic amplitudes resulted in a significant reduction of cutting forces at low rotational/milling speeds (*A* = 5 μm, *n_s_* = 6000 r/min). At small feed per tooth value *f_z_* = 10 μm/r, the superimposed ultrasonic amplitude *A* = 5 μm was more likely to result in lower milling forces as shown in Figure 3c,f,i.

### 3.2. Analysis of Main Effect Trends

The trend of the influence of the interaction between the machining parameters is assessed through the experiment of milling force. Data processing of the test values is required to ensure the results are not due to chance.

The order of the influence of each machining parameter on the milling force was obtained by the Polarization Difference analysis of the test data. The value of polarization difference (PD) is denoted by K. This orthogonal test is a four-factor three-level test with three levels of polarization difference as K_1_, K_2_ and K_3_. The results are shown in Table 5. The decreasing order of the influence of each milling parameter on the milling force components was as follows: 

on *Fx*:f_z_ > *a_p_* > *n_s_* > *A*, 

on *Fy*:f_z_ > *a_p_* > *A* > *n_s_*, 

on *Fz*:f_z_ > *A* > *a_p_* > *n_s_*.

**Figure 4:**
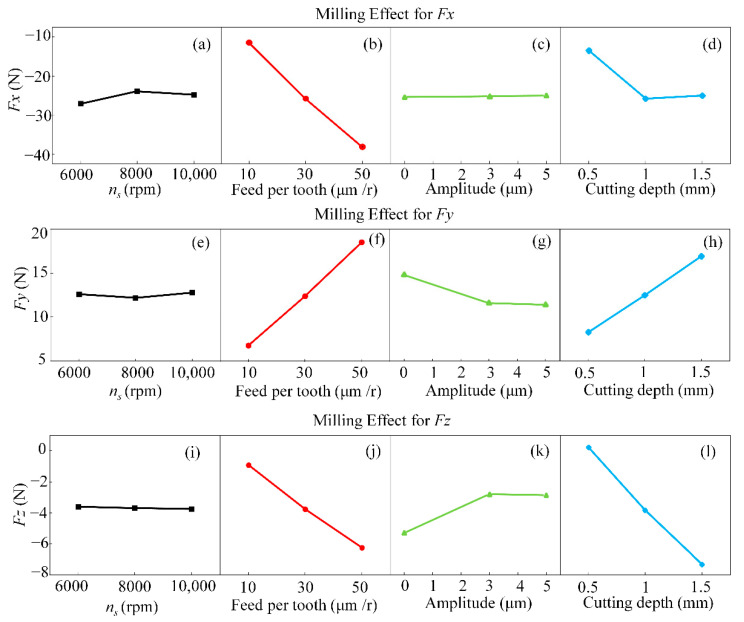
Main effects of various milling parameters on milling forces *Fx*, *Fy* and *Fz*. (**a**,**e**,**i**) Effect of spindle speed on milling forces *Fx*, *Fy* and *Fz* under polarization difference analysis; (**b**,**f**,**j**) Effect of feeds per tooth on milling forces *Fx*, *Fy* and *Fz* under polarization difference analysis; (**c**,**g**,**k**) Effect of ultrasonic amplitude on milling forces *Fx*, *Fy* and *Fz* under polarization difference analysis; (**d**,**h**,**l**) Effect of ultrasonic cutting depth on milling forces *Fx*, *Fy* and *Fz* under polarization difference analysis.

To clarify the effect of machining parameters on milling forces, main effect plots were obtained by Polarization Difference analysis, as shown in Figure 4. These plots were also categorized and discussed to assess the specific effect of each parameter on the cutting force.

It can be seen in Figure 4 that plots of *Fx*, *Fy* and *Fz* exhibit similar patterns with slight quantitative differences. This is because the longitudinal ultrasonic vibration directly affects the longitudinal milling force (*Fz*), and the superimposed torsional ultrasonic vibration indirectly affects the tangential milling force (*Fy*). The main patterns observed in Figure 4g,k imply that the milling force tended to decrease significantly with the ultrasonic amplitude due to the increased ultrasonic amplitude, promoting the contact and separation between the specimen and the tool. It also prolonged the duration of this intermittent milling, reducing the milling force.

In general, aluminum material is softer. When the milling speed is increased, the heat generated by the material in the shear range increases, which reduces the elastic deformation capacity of the aluminum alloy and reduces the shear strength of the material, thus the milling force has a tendency to decrease. An increase in spindle speed will reduce the shear angle and shear area, and the change in shear surface area leads to a reduction in shearing force. In the main effect diagram, due to the high specific strength and specific stiffness of the 2195 Al-Li alloy, increasing the friction between the tool and the material during high-speed milling increases, resulting in a slight increase in milling force. As shown in Figure 4a,e,i. the effect of rotational speed on the milling force of the 2195 aluminum-lithium alloy is not significant because the shearing force decreases and the friction force increases at the same time by increasing the spindle speed. As shown in Figure 4b,d,f–h,l, the increase in shear stress exceeded the strength of tool in contact with the material, so that the milling force increased slightly. Meanwhile, increased milling force was linked to increased feed per tooth and depth of cut. The latter resulted in higher cutting thickness and closer tool contact with the specimen, thus increasing the milling force.

### 3.3. Single-Factor Analysis

**Figure 5:**
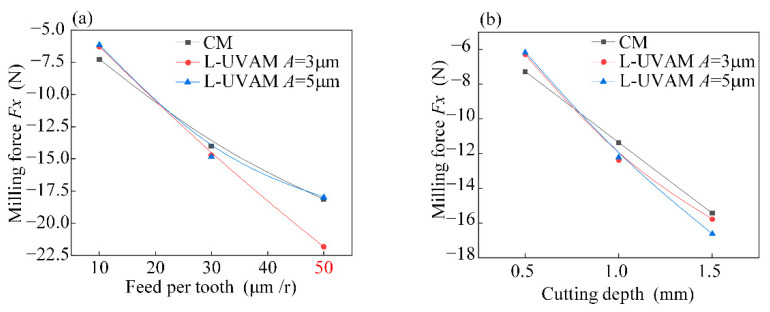
Single-factor analysis of *Fx*. (**a**) Effect of variation of feed per tooth on milling force *Fx*; (**b**) Effect of cutting depth variation on milling force *Fx*.

**Figure 6:**
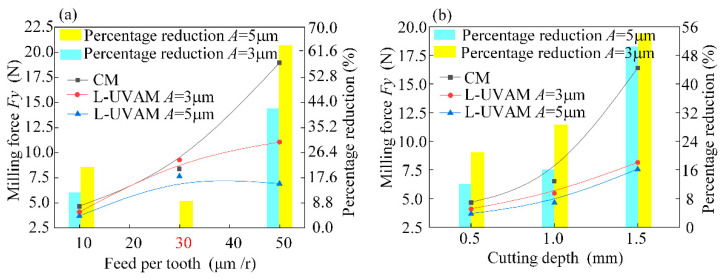
Single-factor analysis of *Fy*. (**a**) Effect of variation of feed per tooth on milling force *Fy*; (**b**) Effect of cutting depth variation on milling force *Fy*.

**Figure 7:**
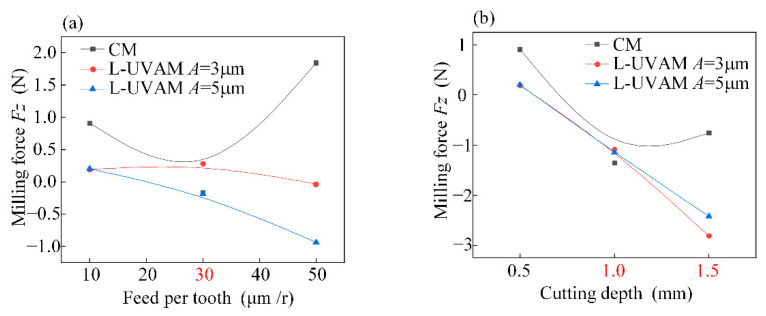
Single-factor analysis of *Fz*. (**a**) Effect of variation of feed per tooth on milling force *Fz*; (**b**) Effect of cutting depth variation on milling force *Fy*.

The effect of rotational speed on milling forces is superimposed on other machining parameters when the interaction effect is considered. However, the polarization difference analysis of the experimental values indicates that the effect of rotational speed on milling forces is negligible, therefore a single factor analysis of the variation of feed per tooth and cutting depth and ultrasonic amplitude can be applied with the available experimental values, as shown in Figure 5, Figure 6 and Figure 7.

Figure 5 shows a single factor analysis of the milling force *Fx*. Increase in feed per tooth and cutting depth causes an increase in milling force. The tool is in closer contact with the workpiece in the x-direction because of the torsional vibration, which results in higher milling force at larger feed and cutting depths.

Figure 6 shows the single factor analysis of milling force *Fy*. Torsional vibration significantly reduces the milling force in the tool feed direction, and the higher amplitude reduces the milling force more effectively. Such a phenomenon is the result of the chip pulling effect and tool separation characteristics caused by torsional vibration. In Figure 6a, an amplitude of 5 mm reduces the milling force by 62% at feed per tooth of 50mm. In Figure 6b, an amplitude of 5 mm reduces the milling force by 54%.

Figure 7 shows the single factor analysis of the milling force *Fz*. As shown in Figure 7a,b, the milling force *Fz* for conventional milling (CM) has a tendency to increase and then decrease followed by an increase due to the increase in feed per tooth and cutting depth; the milling force is minimal at *f_z_* = 30 mm and *a_p_* = 1 mm. The contact-separation characteristic of the longitudinal vibration causes a change in the depth of cut in the vertical direction, which causes the milling force to change to the opposite direction. Higher amplitude leads to higher longitudinal high-frequency shocks and more pronounced contact-separation characteristics.

### 3.4. Milling Force Signal Spectrum Characteristics

**Figure 8:**
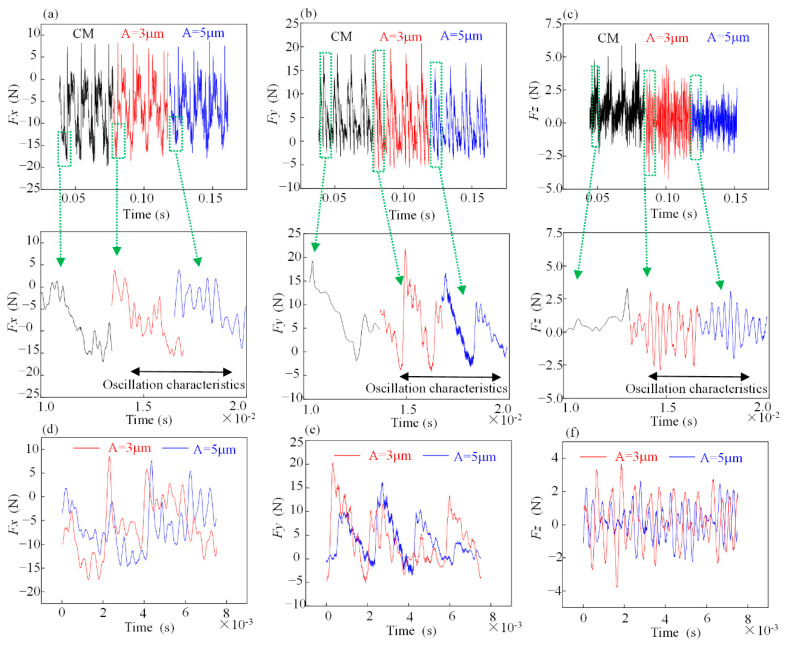
Single-factor test on the milling force signal (*n_s_* = 8000 r/min, *f_z_* = 10 μm, *a_p_* = 0.5 mm). (**a**–**c**) The collected milling force signals *Fx*, *Fy* and *Fz*; (**d**–**f**) Milling force signals of *Fx*, *Fy* and *Fz* at amplitudes *A* = 3 μm and *A* = 5 μm during one cycle of tool tip contact with the workpiece.

Figure 8 shows the test values of milling forces (*Fx*, *Fy* and *Fz*) for different amplitudes with the same other milling parameters (*n_s_* = 8000 r/min, *f_z_* = 10 μm/r and *a_p_* = 0.5 mm).

As shown in Figure 8a,b, the ultrasonic vibration with amplitude *A* = 3 μm caused a slight increase in the peak milling force, but its mean value decreased. This was due to the superimposed effect of the overall system vibration frequency and ultrasonic vibration resulting in an increase in the peak cutting force. However, the ultrasonic vibration caused a change in contact and separation between the tool and the test piece, resulting in a decrease in the mean value of the peak cutting force. At an amplitude *A* = 5 μm, the peak and mean values of the transverse milling forces (*Fx* and *Fy*) were reduced. That is, the effect of ultrasonic vibration exceeded that produced by the equipment resonance. A longer separation window between the tool and the specimen reduced both the peak and mean values of the milling forces. Thus, the longitudinal ultrasonic vibration is one of the main factors in the change of tool trajectory, as shown in Figure 8c. Compared with conventional milling, the peak milling force (*Fz*) of the test at *A* = 5 μm was smoother and less volatile, which can be considered as the result of the “sinusoidal” variation of the tool motion. When the amplitude was increased, as shown in Figure 8d–f, the increase in the amplitude led to a backward shift in the peak test milling force.

**Figure 9:**
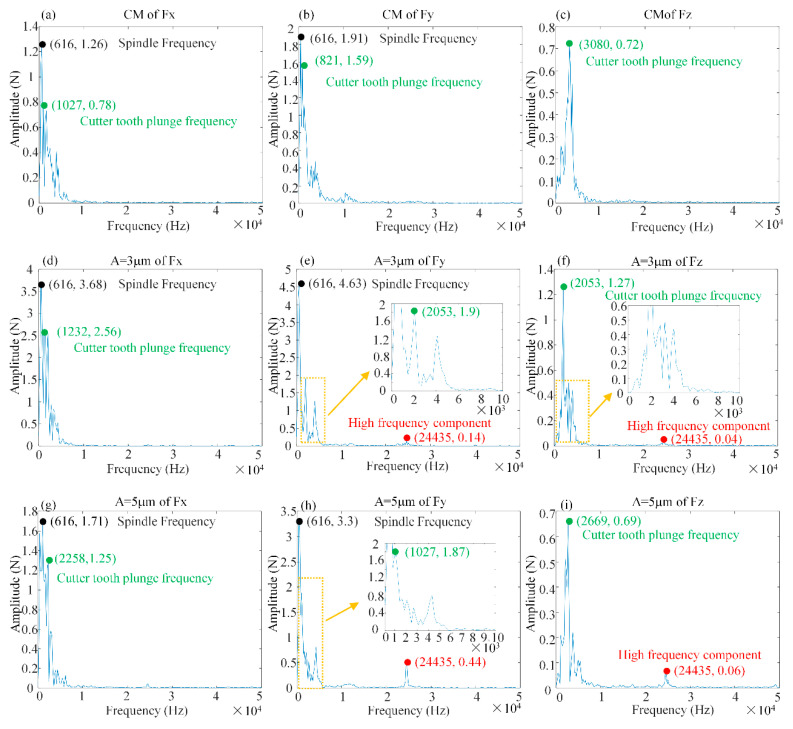
Milling force spectrum characteristics (*n_s_* = 8000 r/min, *f_z_* = 10 μm, *a_p_* = 0.5 mm). (**a**–**c**) Spectrum analysis of milling forces *Fx*, *Fy* and *Fz* for conventional machining; (**d**–**f**) Spectrum analysis of milling forces *Fx*, *Fy* and *Fz* for ultrasonic vibration amplitude *A* = 3 μm; (**g**–**i**) Spectrum analysis of milling forces *Fx*, *Fy* and *Fz* for ultrasonic vibration amplitude *A* = 5 μm.

To investigate the effect of ultrasonic vibration with different amplitudes on the spectral characteristics of milling forces, the fast Fourier transform (FFT) was performed for conventional milling, as shown in Figure 9a–c, and ultrasonic milling was performed with ultrasonic amplitudes *A* = 3 μm in Figure 9d, Figure 9e and Figure 9f, and *A* = 5 μm in Figure 9g, Figure 9h and Figure 9i, respectively.

The spindle rotation frequency in conventional (CM) and ultrasonic-assisted milling (L-UVAM) did not change with increasing amplitude. The tool cutting frequencies of *Fx* and *Fy* under normal milling were 1027 Hz and 821 Hz, respectively. By applying ultrasonic vibration with a 3 mm amplitude, the tool cutting frequencies of *Fx* and *Fy* were increased to 1232 and 2053 Hz, respectively. At an ultrasound amplitude of 5 μm, the tool cutting frequency of *Fx* continued to increase to 2258 Hz, while *Fy* was reduced by the effect of torsional vibration, resulting in a stable tool cutting frequency of 1027 Hz. The tool plunge frequency of *Fz* was reduced by applying ultrasonic vibration. In addition, the ultrasonic vibration with applied longitudinal torsion induced the high-frequency components of *Fy* and *Fz*.

### 3.5. Surface Morphology Analysis

**Figure 10:**
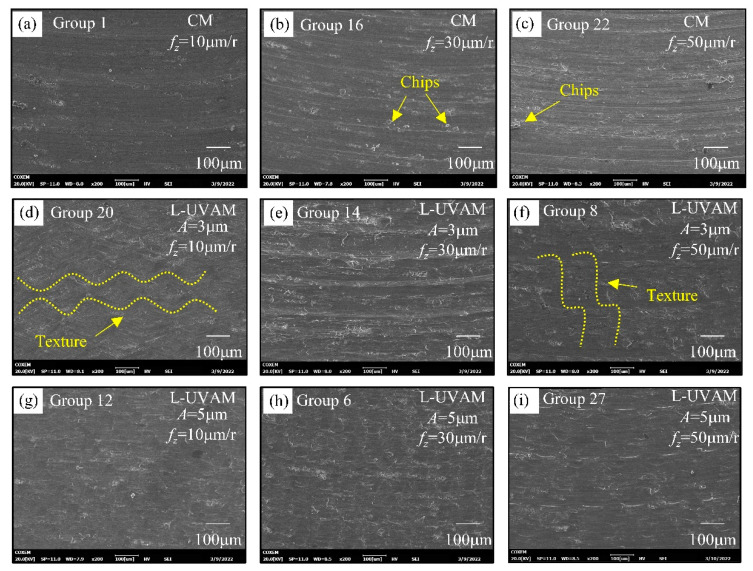
Effect of feed per tooth on the surface morphology of Conventional Milling (CM) and Longitudinal Torsion - Ultrasonic Vibration Assisted Milling (L-UVAM). (**a**–**c**) Effect of increasing feed per tooth on surface topography in CM; (**d**–**f**) Effect of ultrasonic vibration amplitude *A* = 3 μm on surface morphology; (**g**–**i**) Effect of ultrasonic vibration amplitude *A* = 5 μm on surface morphology.

The magnitude of the milling force has a direct correlation to the surface topography. Figure 10 shows the microscopic morphology images of CM and L-UVAM machined surfaces observed by scanning electron microscopy. As the images of the surface morphology in Figure 10a–c show, there are obvious tool marks on the CM machined surface. The increase in feed per tooth increases the milling force, the chip residue on the surface increases, and the lateral plastic flow of the machined surface material becomes more severe, resulting in significant scraping. As shown in Figure 10d–f, the surface topography is changed due to the reduction of milling force at *f_z_* = 10 μm and *A* = 3 mm. The torsional vibration smooths out the tool marks and creates a “sinusoidal” texture on the surface in the direction of the feed. As shown in Figure 10g–i, increasing amplitude causes higher density of traces left by the tool trajectory, surface smearing effect is more obvious, in addition, the impact load increases to increase the material removal rate and reduce the surface chips. Ultrasonic vibration creates a unique texture on the workpiece, which efficiently removes chips and also reduces surface roughness, ultimately improving the surface quality of the workpiece and frictional wear and other characteristics.

## 4. Conclusions

This paper presents the results of a study of the effect of two machining processes, CM and L-UVAM, on the milling forces of 2195 Al-Li alloys with varying machining parameters. The interaction effect analysis was combined with the single factor analysis to analyze the variation of milling force. The effects of feed per tooth and depth of cut on milling forces were analyzed, as well as changes in force signals and spectra in ultrasonic machining, and changes in surface topography due to changes in milling forces. The study lays a theoretical foundation for further research and optimization of aluminum-lithium alloys in the machining milling process, and provides some experimental basis for cold machining of aluminum-lithium alloys. The following main conclusions were obtained from the investigation:

(1) In general, cutting depth directly affects the amount of milling force. Exploring the effects of milling speed, feed per tooth and ultrasonic amplitude on the interaction of milling forces revealed that higher speeds with *f_z_* = 50 μm and lower speeds with *f_z_* = 10 μm both have a significant effect on milling forces *Fx* and *Fy*. Both L-UVAM milling with *A* = 5 μm at *f_z_* = 10 μm and CM machining with *f_z_* = 50 μm decreased *Fz*.

(2) Longitudinal torsional vibration significantly reduces the milling force, and the reduction increases with increasing feed per tooth and cutting depth. Compared to CM milling, the milling force *Fy* can be reduced by up to 40% for *f_z_* = 50 mm and up to 50% for *a_p_* = 1.5 mm at *A* = 3 mm. The maximum reduction of milling force *Fy* is 62% for *f_z_* = 50 mm and 55% for *a_p_* = 1.5 mm at *A* = 5 mm.

(3) The L-UVAM milling process through the tool motion trajectory analysis can be known longitudinal torsion of ultrasonic vibration to the tool tip from the original two-dimensional motion to three-dimensional motion, longitudinal vibration and torsional rotation on the tool superposition to make the collected milling force signal to change. L-UVAM caused the cutting force signal to exhibit oscillation characteristics; with an increase in the ultrasonic amplitude, the periodic contact separation of the tool became more pronounced, delaying the peak time of the milling force. L-UVAM caused significant changes in the low-frequency components of the milling force signal spectrum (spindle frequency and tool plunge frequency, as well as other multipliers), as well as high-frequency components in the spectrum of milling forces *Fy* and *Fz*.

(4) The reduction of milling force has an important effect on surface topography, and longitudinal torsional ultrasonic vibration can not only reduce the milling force but also change the surface topography characteristics. In CM machining, the milling force increases with the increase of feed per tooth, which leads to serious lateral plastic flow of material on the machined surface of the Al-Li alloy, and the surface scratches are obvious, the chip accumulation is serious, and the surface morphology is not regular. L-UVAM machining reduces the milling force, so it improves the surface topography. The high frequency impact of longitudinal vibration increases material removal rates, reduces chip build-up on the surface, and the variable cutting depth created by the oscillating feature creates texture on the surface. Torsional vibration reduces milling forces in the feed direction and has a smoothing and “ironing” effect, reducing surface scratches.

In conclusion, CM and L-UVAM machining of the 2195 Al-Li alloy has collected a large number of results on milling forces and some regular conclusions have been obtained. This paper fills some gaps in the field of longitudinal torsional ultrasonic vibration milling processing for 2195 Al-Li alloys, but more research is worthy of deeper investigation, for example, whether the test parameters could be applied when milling 2195 Al-Li alloys at low speed. As the surface integrity of different materials under different processing methods is greatly affected, the surface morphology is associated with the surface integrity, and further research is needed to demonstrate whether the milling force has an effect on the residual stress, surface roughness and other factors. In the future, research on 2195 aluminum-lithium alloys should consider such content.

## Figures and Tables

**Table 1:** Chemical composition of the 2195 alloy (wt%).

Cu	Li	Mg	Zr	Al	Ag	Fe	Ti	Si
4.06	0.97	0.67	0.1	Margin	0.41	0.03	0.04	0.02

**Table 2:** Mechanical characteristics of the 2195 alloy.

Density *ρ*/g (cm^−3^)	Tensile Strength *Rm* (Mpa)	Yield Stress *R_po2_* (MPa)	Post-Break Elongation *A (*%)	Fracture Toughness *K_Ic_* (MPa)	Elastic Modulus *E* (GPa)
2.72	552	517	8.0	31	76.0

**Table 3:** Orthogonal test scheme.

Level	Processing Parameters			
	*n_s_* (r/min)	*f_z_* (μm /r)	*a_p_* (mm)	*A* (mm)
1	6000	10	0.5	0
2	8000	30	1	3
3	10,000	50	1.5	5

**Table 4:** The orthogonal test results on the milling force of the 2195 Al-Li alloy.

No.	*n_s_* (rpm)	*f_z_* (μm /r)	*A* (μm)	*a_p_* (mm)	*Fx* (N)	*Fy* (N)	*Fz* (N)
1	6000	10	0	0.5	−7.27	4.65	0.91
2	6000	10	3	1	−12.38	5.46	−1.09
3	6000	10	5	1.5	−16.63	7.56	−2.42
4	6000	30	0	1	−25.97	13.79	−3.57
5	6000	30	3	1.5	−38.13	13.47	−7.78
6	6000	30	5	0.5	−14.86	7.65	−0.19
7	6000	50	0	1.5	−63.95	30.13	−11.64
8	6000	50	3	0.5	−21.83	11.06	−0.04
9	6000	50	5	1	−42.46	19.96	−6.54
10	8000	10	0	1	−11.37	6.51	−1.36
11	8000	10	3	1.5	−15.78	8.15	−2.81
12	8000	10	5	0.5	−6.17	3.68	0.20
13	8000	30	0	1.5	−34.14	14.02	−7.50
14	8000	30	3	0.5	−14.73	9.29	0.28
15	8000	30	5	1	−24.59	11.03	−4.10
16	8000	50	0	0.5	−18.14	18.95	1.84
17	8000	50	3	1	−36.6	16.02	−6.43
18	8000	50	5	1.5	−52.89	22.50	−12.44
19	10,000	10	0	1.5	−15.44	16.39	−0.76
20	10,000	10	3	0.5	−6.29	4.09	0.19
21	10,000	10	5	1	−12.22	4.65	−1.15
22	10,000	30	0	0.5	−14.01	8.39	−0.17
23	10,000	30	3	1	−29.1	15.17	−3.64
24	10,000	30	5	1.5	−37.68	19.06	−7.24
25	10,000	50	0	1	−38.03	20.07	−6.74
26	10,000	50	3	1.5	−52.36	20.86	−13.29
27	10,000	50	5	0.5	−17.99	6.91	−0.94

**Table 5:** Analysis of the influence of milling parameters.

PD	*n_s_* (rpm)			*f_z_* (μm /r)			*A* (μm)			*a_p_* (mm)		
	*Fx*	*Fy*	*Fz*	*Fx*	*Fy*	*Fz*	*Fx*	*Fy*	*Fz*	*Fx*	*Fy*	*Fz*
K1	−27.1	12.6	−3.6	−11.5	6.8	−0.9	−25.4	14.8	−5.3	−13.5	8.3	0.2
K2	−23.9	12.3	−3.6	−25.9	12.4	−3.8	−25.2	11.5	−2.8	−25.9	12.5	−3.9
K3	−24.8	12.8	−3.8	−38.3	18.5	−6.3	−25.1	11.4	−2.9	−36.4	16.9	−7.3
Range Error	3.2	0.60	0.19	26.7	11.7	5.3	0.4	3.3	2.5	22.9	8.6	0.5

## Data Availability

See the supplement file for details.

## Supplementary Materials

The following supporting information can be downloaded at: https://www.mdpi.com/article/10.3390/ma15072508/s1, Figure S1: Effect of milling force on surface topography No. 1; Figure S2: Effect of milling force on surface topography No. 2; Figure S3: Effect of milling force on surface topography No. 3; Figure S4: Effect of milling force on surface topography No. 4; Figure S5: Effect of milling force on surface topography No. 5; Figure S6: Effect of milling force on surface topography No. 6; Figure S7: Effect of milling force on surface topography No. 7; Figure S8: Effect of milling force on surface topography No. 8; Figure S9: Effect of milling force on surface topography No. 9.
