# Supplementary material for: Experimental Study on Ultrasonic-Assisted End Milling Forces in 2195 Aluminum-Lithium Alloy
Source: Materials (Basel). 2022 Mar 29;15(7):2508. doi: 10.3390/ma15072508 (PMC8999360; doi:10.3390/ma15072508)
Supplement: Supplementary file 1 [file materials-15-02508-s001.zip › materials-1630217-supplementary.pdf]

materials

Supplementary Materials

Experimental Study on Ultrasonic-Assisted End Milling Forces in 2195 Aluminum-Lithium Alloy

Hongtao Wang [1], Shaolin Zhang [1] and Guangxi Li [2,*]

[1] School of Mechanical and Power Engineering, Zhengzhou University, Zhengzhou 450001,China; wanghongtaozzu@163.com (H.W.); zhangshaolin@zzu.edu.cn (S.Z.)
[2] Henan Engineering Research Center for Ultrasonic Technology and Application, Pingdingshan University, Pingdingshan 467000, China
* Correspondence: lgxleaning@163.com

Citation: Wang, H.; Zhang, S.; Li, G. Experimental Study on Ultrasonic-Assisted End Milling Forces in 2195 Aluminum-Lithium Alloy. *Materials* **2022**, *15*, 2508. https://doi.org/10.3390/ma15072508

Academic Editors: Paweł Twardowski and Michal Wieczorowski

Received: 23 February 2022
Accepted: 20 March 2022
Published: 29 March 2022

Publisher's Note: MDPI stays neutral with regard to jurisdictional claims in published maps and institutional affiliations.

Copyright: © 2022 by the authors. Licensee MDPI, Basel, Switzerland. This article is an open access article distributed under the terms and conditions of the Creative Commons Attribution (CC BY) license (http://creativecommons.org/licenses/by/4.0/).

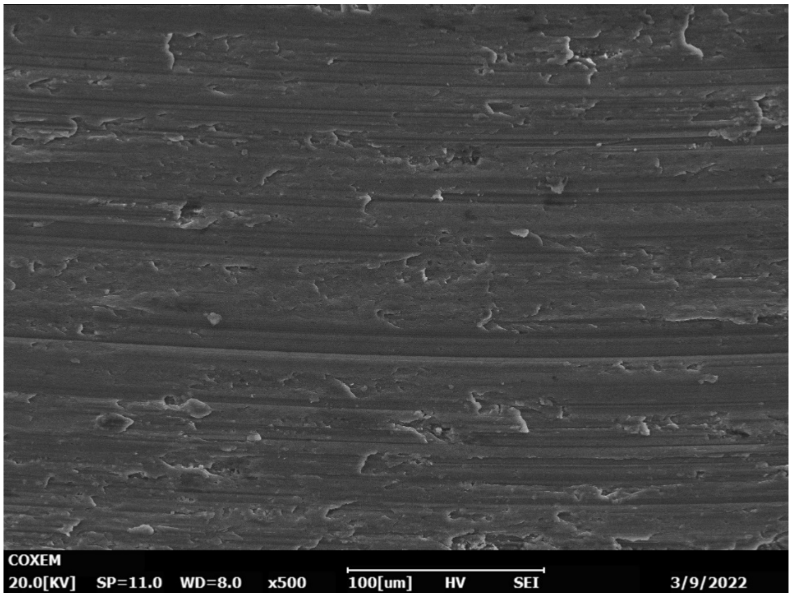

Figure S1. Effect of milling force on surface topography No. 1 .

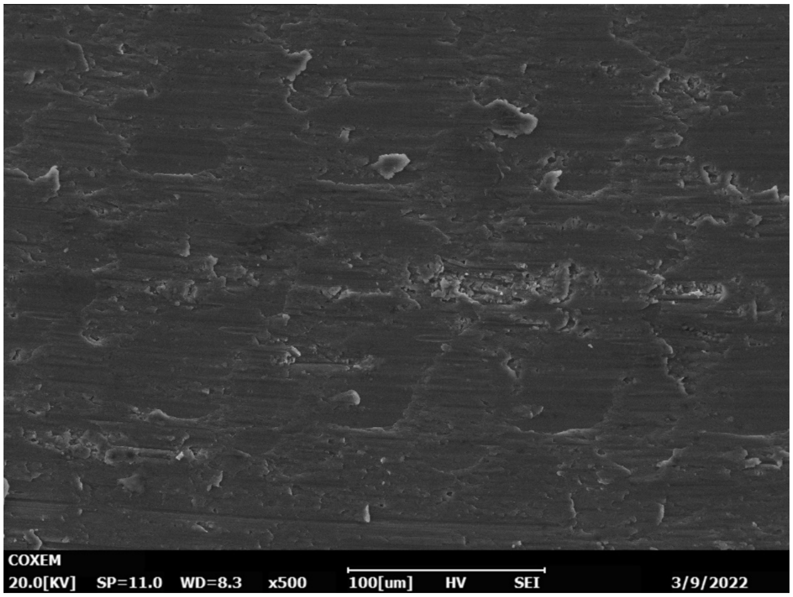

Figure S2. Effect of milling force on surface topography No. 2.

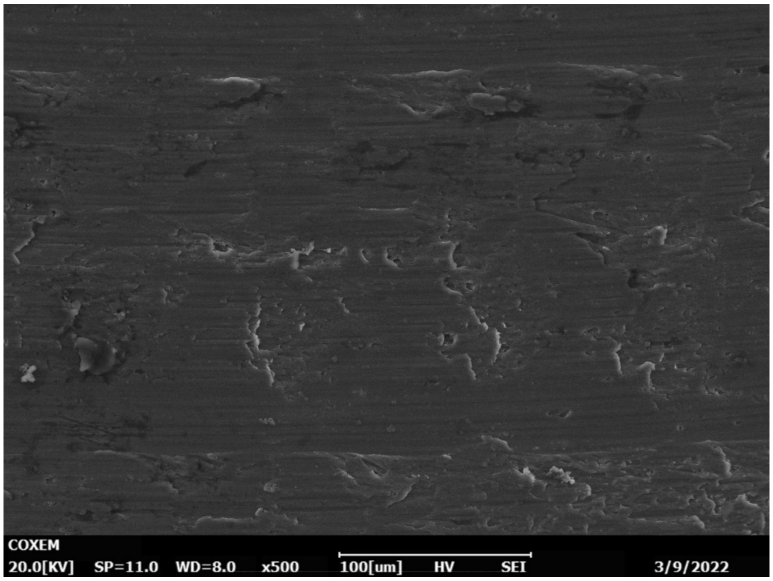

Figure S3. Effect of milling force on surface topography No. 3.

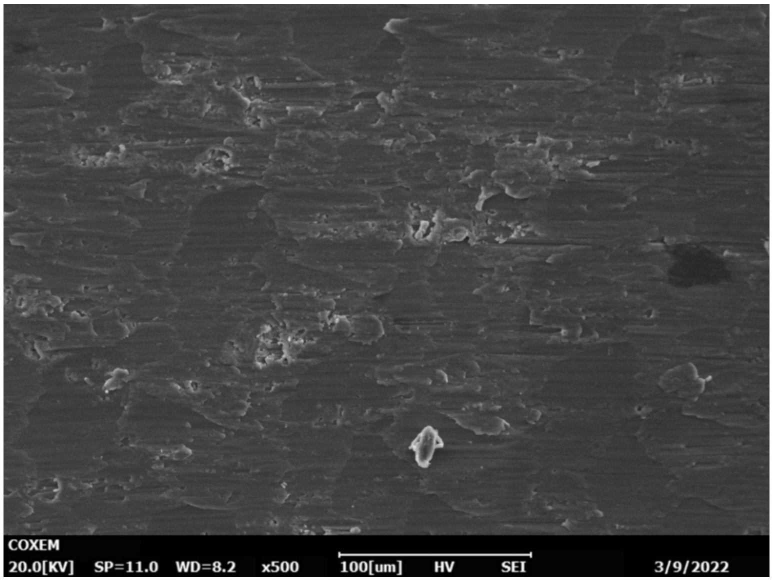

Figure S4. Effect of milling force on surface topography No. 4.

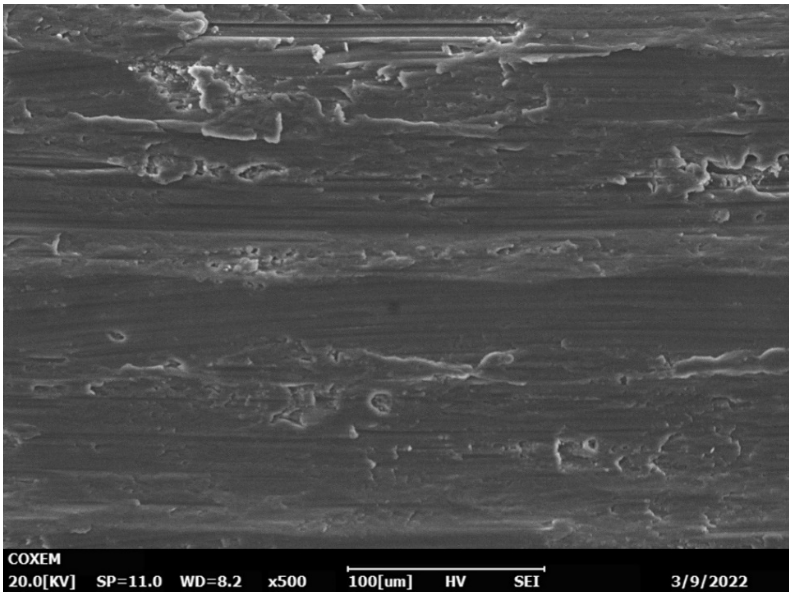

Figure S5. Effect of milling force on surface topography No. 5.

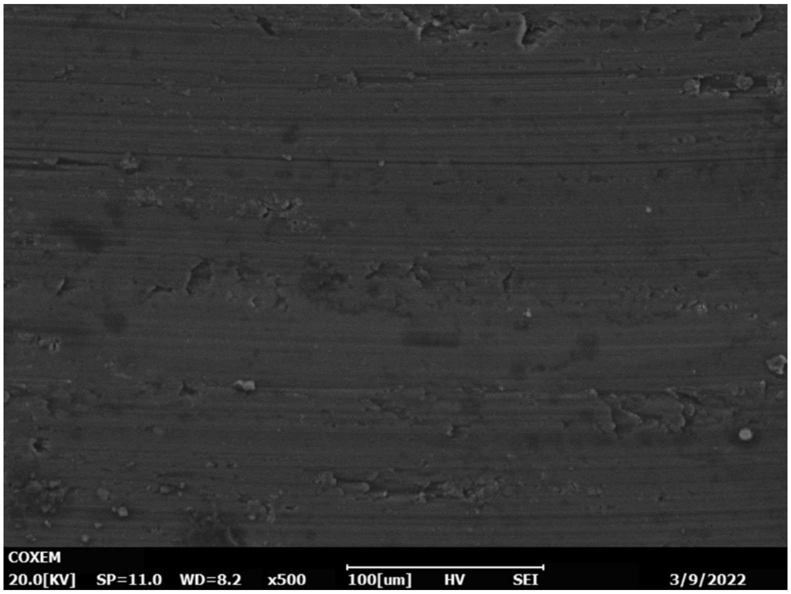

Figure S6. Effect of milling force on surface topography No. 6.

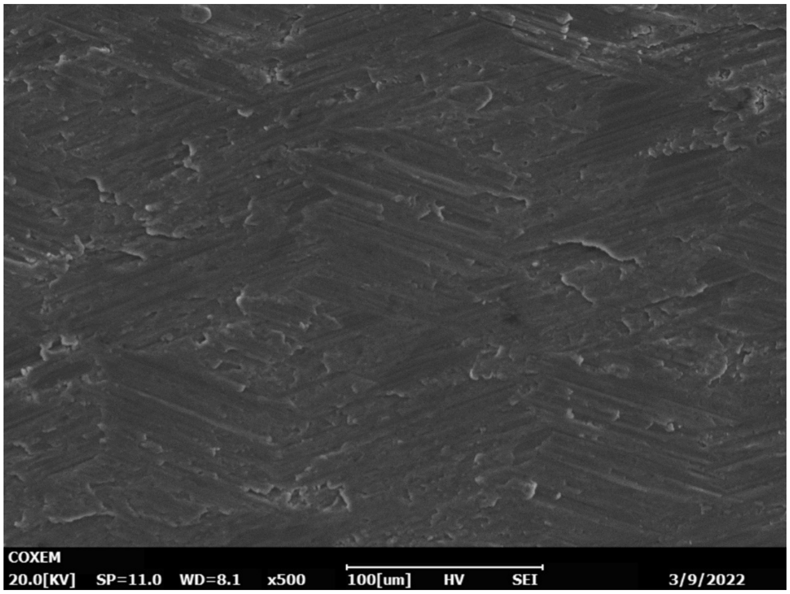

Figure S7. Effect of milling force on surface topography No. 7.

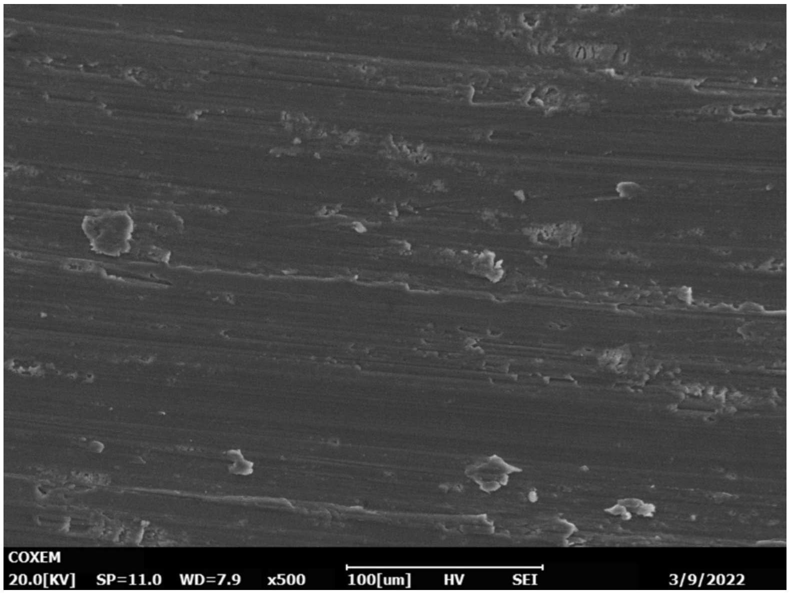

Figure S8. Effect of milling force on surface topography No. 8.

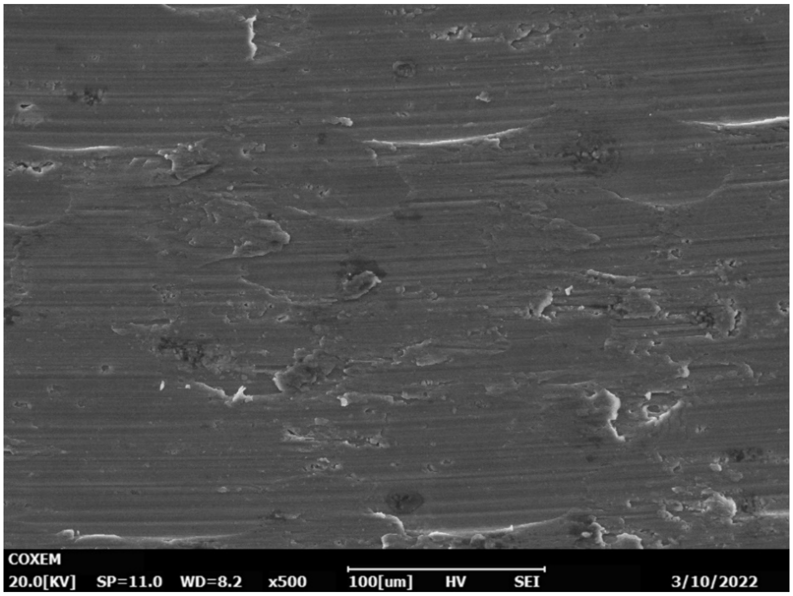

Figure S9. Effect of milling force on surface topography No. 9.
